# Death of Dr. Drake

**Published:** 1853-01

**Authors:** 


From the Cincinnati Gazette.

*Death of Dr. Drake.*

In Dr. Daniel Drake, Cincinnati has lost one of its oldest pioneers—an eminent citizen—a most distinguished physician—a man of science, and a consistent Christian. Such a loss has no reparation, but in the hope that, guided and enlightened by such an example, another generation may raise up others like him. For ourselves, we can only lament the star that is set, on this hemisphere, to rise no more.

In the brevity of these few lines there is only room to record some of the epochs in his life, some dates in his great public services, and note something of his peculiar character.

Dr. Drake was born in 1784, in New Jersey, whence his father moved to Kentucky. It was in Mason county, Ky., in the pioneer society, and in rural scenes, to which, in after life, he often referred, that he received his early impressions of men and Nature. Of both he was ever after an ardent student. With man, both social and physical—with Nature, both scientific and picturesque—all his pursuits and tastes led him to a familiar knowledge. With a peculiar aptitude for such tastes and pursuits, formed amid the scenery of woods and fields, and in the simplicity of primitive society, it was but natural that he should turn his attention to the study of medicine—that science which, more than any other, led to a familiar acquaintance with the objects and subjects upon which his mind dwelt. Accordingly, he commenced the study of medicine with Dr. Goforth, of Cincinnati, in his 16th year, 1800. He was, therefore, at his death, a physician *half a century in practice*, and though occasionally absent, both as teacher and traveller, never in that time, lost his citizenship in Cincinnati.

In constitution active, energetic and sanguine, he early became imbued with that best form of ambition, the ambition of being a public benefactor in the walks of Science, Letters, Utility, and Benevolence. It is vain here to attempt even an outline of his brilliant services in that career. Many of them have left an enduring impress on society, and become monuments to the reality of his benefactions, even when unremembered by those who profit by the results.

While a very young man, he became one of the active founders of several scientific and benevolent Societies, whose objects were the cultivation of Science and the public good. Before these, he often delivered lectures, or essays, on topics of Natural History, Medicine, or Social Action. One of these was published in the form of "Sketches of Cincinnati," which soon after became the foundation of a more elaborate work, entitled "Picture of Cincinnati," including a Natural and Statistical View of the Miami Country, published in 1815. This made him known both in Europe and America, and was one of the first things which drew the attention of the public to this town and section of the country.

So full of research and so accurate was this natural history of the Miami country, and of the locality of Cincinnati, that nothing substantial has been added to the account since. The "discourse" delivered before the Medical Society last winter, was his own proper sequel to that work, filled with reminiscences of our early progress and society.

The Medical College of Ohio is the next monument to his labors. The charter of that institution was passed January, 1819, and was passed by the Legislature on his own personal application. During many years subsequent he was one of its professors. During his whole after life, it occupied his thoughts and his cares; and in its cause, by overtasking an ever-active brain, he at last died.

Separated for a time from that, by changes incident to medical politics, he became a professor in the Lexington School, in the period of its greatest prosperity; and in recent years, in the Louisville School, which rapidly rose to unprecedented success.

In the meantime, 1836–7, he was the founder of a new Medical School in Cincinnati, attached to Cincinnati College. This school flourished in the brief period of its existence, but was given up for the want of an endowment. It was about the same period that Dr. Drake, in the same public spirit which ever animated him, became one of the earliest promoters of our railway system.

He was one of the first, if not the first, to propose the great Southern Line to Charleston, S. C. He was one of the active delegates to the Knoxville Convention, where were commenced the first efforts for the Railway Lines (soon to be completed) between the Ohio Valley and the Southern Atlantic. For the last six or seven years his hours of unprofessional service have been devoted ardently and intently to his last great work on the Diseases and Climatology of the Mississippi Valley. One volume of this, occupying near a thousand pages, has already been published, and it is no exaggeration of its merits to say, that it stands, so far, the greatest work of American science. It is appreciated abroad and will be at home. It was in the attempt to finish this work—the only remaining ambition of his life—now left, like a creation of human hopes, a broken structure—that he fell in the harness, and died on the Battle-field of Life.

From his domestic life we lift only enough of the veil to disclose the strength of affection and friendship, which all who knew loved and valued above all public fame. It wearied in no labor of love—failed in no hour of adversity, nor paled before any other light.

In 1806, he married Harriet Sisson, a niece of Gen. Mansfield, then a resident of Ohio. With her he lived near twenty years, with a conjugal devotion, which seemed equal to that pictured in romance, and from her death lived a widower, ever sorrowing over her memory.

Three of his children, now with families, were his constant care and his ever-present thought; with his two daughters he lived at his death, and as he bade the world farewell, bade them not sorrow over him; for long since he had surrendered himself to his and their Redeemer.

Here we finish this brief tribute to friendship and worth—less than half we would—more than he needs; for henceforth,

> "Entertain him all the saints above,
> In solemn troops and sweet societies,
> That sing, and singing, in their glory move."

